# Anti-RBD IgA and IgG Response and Transmission in Breast Milk of Anti-SARS-CoV-2 Vaccinated Mothers

**DOI:** 10.3390/pathogens11030286

**Published:** 2022-02-24

**Authors:** Felicia Trofin, Eduard Vasile Nastase, Luminita Smaranda Iancu, Daniela Constantinescu, Corina Maria Cianga, Catalina Lunca, Ramona Gabriela Ursu, Petru Cianga, Olivia Simona Dorneanu

**Affiliations:** 1 Microbiology Department, University of Medicine and Pharmacy “Grigore T. Popa”, 700115 Iasi, Romania; felicia.trofin@umfiasi.ro (F.T.); catalina.lunca@umfiasi.ro (C.L.); ramona.ursu@umfiasi.ro (R.G.U.); olivia.dorneanu@umfiasi.ro (O.S.D.); 2 Clinical Hospital of Infectious Diseases “Sf. Parascheva”, 700116 Iasi, Romania; eduard-vasile.nastase@umfiasi.ro; 3 Infectious Diseases Department, University of Medicine and Pharmacy “Grigore T. Popa”, 700115 Iasi, Romania; 4 National Institute of Public Health, Iasi Regional Center for Public Health, 700465 Iasi, Romania; 5 Immunology Department, University of Medicine and Pharmacy “Grigore T. Popa”, 700115 Iasi, Romania; daniela.constantinescu@umfiasi.ro (D.C.); corina.cianga@umfiasi.ro (C.M.C.); 6 Immunology Laboratory, “Sf. Spiridon” Clinical Hospital, 700111 Iasi, Romania

**Keywords:** immunoglobulins, SARS-CoV-2, COVID-19, vaccine, breast milk

## Abstract

The appearance of the severe acute respiratory syndrome virus-2 (SARS-CoV-2) has had a significant impact on the balance of public health and social life. The data available so far show that newborns and young children do not develop severe forms of COVID-19, but a small proportion of them will still need hospitalization. Even though young children represent an important vector of the infection, vaccination at such a young age was not yet considered. Thus, the question of whether potentially protective antibodies against SARS-CoV-2 could be provided to them via breast milk or across the placenta, as “passive immunity”, still stands. Materials and Methods: Between January–July 2021, we have conducted a prospective study that aimed to measure the immunoglobulin (Ig) A and IgG anti-SARS-CoV-2 titers in the breast milk of 28 vaccinated lactating mothers, sampled at 30 and 60 days after the second dose of the anti-SARS-CoV-2 Pfizer or Moderna mRNA vaccines. Anti-RBD reactive IgA and IgG antibodies were detected and quantified by a sandwich enzyme-linked immunosorbent assay. Results: Anti-RBD IgA and IgG were present in all breast milk samples, both in the first and in the second specimens, without a significant difference between those two. The anti-RBD IgA titers were approximately five-times higher than the anti-RBD IgG ones. The anti-RBD IgA and IgG titers were correlated with the infants’ age, but they were not correlated with the vaccine type or mother’s age. The anti-RBD IgA excreted in milk were inversely correlated with the parity number. Conclusions: Anti-SARS-CoV-2 IgA and IgG can be found in the milk secretion of mothers vaccinated with mRNA vaccines and, presumably, these antibodies should offer protection to the newborn, considering that the antibodies’ titers did not decrease after 60 days. The antibody response is directly proportional to the breastfed child’s age, but the amount of anti-RBD IgA decreases with the baby’s rank. The antibody response did not depend on the vaccine type, or on the mother’s age.

## 1. Introduction

The appearance of the severe acute respiratory syndrome virus-2 (SARS-CoV-2) changed the path of our lives, having a significant impact on the balance of public health and social life [1].

It was shown that the SARS-CoV-2 infection leads to an immune response that also generates neutralizing antibodies which target the receptor-binding domain or other regions of the protein [2,3,4]. Importantly, mucosal antibodies of the IgA type can also be detected after an infection [5,6]. The development of anti-SARS-CoV-2 vaccines seems to restore the world’s stability and hope [7]. COVID-19 vaccines are shown to be effective against severe disease, including those caused by the Delta [8] variant and, to a certain level, the Omicron variant [9].

The results of the clinical trials for the two available mRNA anti-SARS-CoV-2 vaccines became readily available. The Pfizer BNT162b1 vaccine was the first to demonstrate its ability to mount both a T helper 1 immune response and a humoral one [10], followed by the results demonstrating that the Moderna vaccine is at least equally effective in terms of efficacy and safety [11] or antibody production [12]. Many more studies supported these data, some also pointing out the production of both immunoglobulins G (IgG) and A (IgA) in the serum of vaccinated adults [13].

To the best of our knowledge, a limited number of studies [14,15,16,17,18,19,20,21,22] detected the presence of IgA and IgG in human milk after SARS-CoV-2 vaccination, but only few aimed at quantifying these antibodies [23].

Even though there are no available published data regarding the impact of COVID-19 neonates in Romania, we can refer to data published in China [24] and the USA [25], which shows incidences lower than 1%. Over 90% of all the Chinese patients were asymptomatic, mild, or moderate cases [20]; hence, a small proportion required hospitalization, while a total of three deaths have been reported in the USA as of 18 April 2020 [25,26]. Nonetheless, infected children seem to represent an important vector of viral spreading [27].

To this date, neither the FDA (Food and Drug Administration) [28] nor the EMA (European Medicines Agency) [29] have considered the administration of the COVID-19 vaccines at such a young age; hence, the protection of the newborn by maternal protective antibodies is a matter of ongoing debate.

The mechanism of passive immunity transfer from mother to offspring is different among mammalian species. In humans, as in rabbits, the transfer of passive immunity from mother to young is performed mostly by the FcRn (neonatal Fc Receptor)-mediated transport of IgG across the placenta [30]. On the other hand, IgA is transferred to the newborn by milk, and it is meant to defend the mucous membranes and to protect against enteric infections [15,31], but with no possibility to be transferred further across these barriers. In fact, the main immunoglobulin in the human milk secretion is IgA, with a concentration of 32 g/L, accounting for cca 90% of the total milk immunoglobulins, while IgG concentration is only 50 mg/L and slightly higher (1 g/L) in the colostrum of non-immunized mothers [32,33], with a different distribution of the four IgG subclasses than in plasma [34], consistent with their FcRn-dependent differences in half-lives [35]. The explanation of this important IgA/IgG disproportion lies in the role of FcRn within the human mammary gland epithelial cells, acting rather as a recycling receptor [36].

The aim of our study was to detect the presence of anti-SARS-CoV-2 IgA and IgG in human breast milk, to measure their concentrations, and to assess if any of the features considered for the characterization of our volunteers might influence the milk antibody titers.

## 2. Results

The study group consisted of 26 lactating mothers aged between 29 and 37 years old. Three (11.5%) of the mothers received the Moderna mRNA-1273 vaccine and the rest of them received the Pfizer BNT162b2 vaccine. The age of the breastfed infants varied between 2 and 35 months at the time of vaccine inoculation, between 3 and 36 months at the first specimen collection, and between 4 and 37 months at the second one. For 17 (65.4%) women, the breastfed baby was their first child; for 8 (30.8%) of them, the baby was the second child, and for 1 (3.8%) the baby was the third child. Autoimmune thyroiditis was the most frequent co-morbidity, encountered in seven (26.9%) participants.

**Figure 1:**
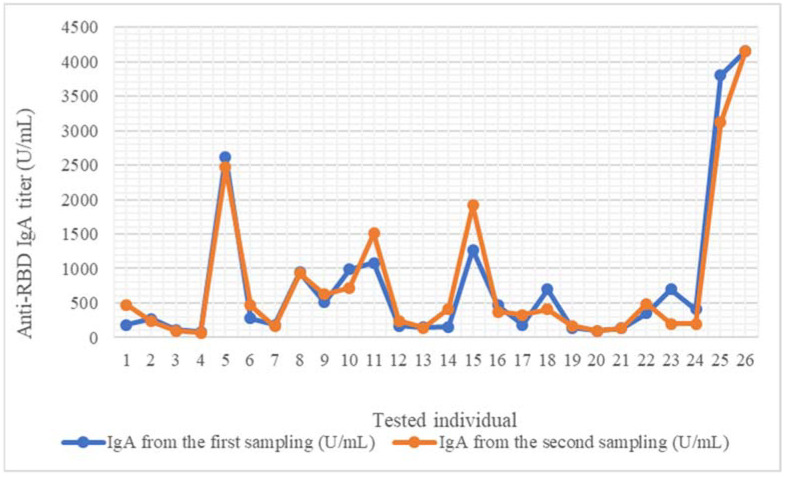
Anti-RBD IgA titer evolution in 30-day intervals for each sampled mother. IgA, first sample (day 30 after second anti-SARS-CoV-2 vaccine). IgA, second sample (day 60 after second anti-SARS-CoV-2 vaccine).

Anti-RBD IgA antibodies were present in all 52 breast milk samples, both in the first and in the second specimens. The antibody titers varied between 84.872 U/mL and 4161.707 U/mL, and between 64.435 U/mL and 4159.966 U/mL for the first and second sample, respectively. Fifteen (57.7%) mothers had a higher IgA titer in the first specimen than the second one, two (7.7%) elicited approximately the same results in both samples, and for nine of them (34.6%), the IgA titer was higher in the second sample (Figure 1). The average for the first sampling was 774.96 U/mL, and for the second one, the average was 774.1 U/mL.

**Figure 2:**
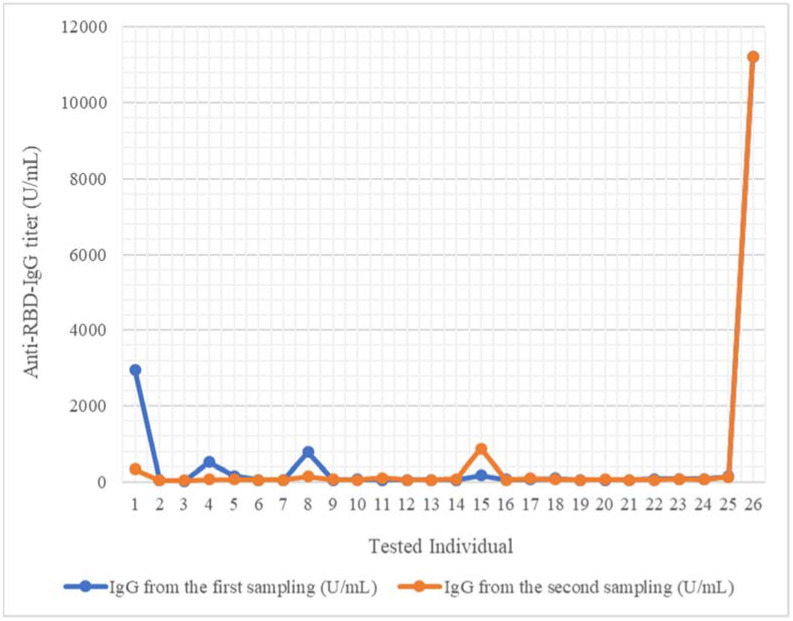
Anti-RBD IgG titer evolution in 30 days interval, in each sampled mother. IgG, first sample (day 30 after second anti-SARS-CoV-2 vaccine). IgG, second sample (day 60 after second anti-SARS-CoV-2 vaccine).

All mothers excreted anti-RBD IgG in breast milk. The titers ranged between 22.962 U/mL and 11,206.348 U/mL for the first specimen, and between 39.435 U/mL and 11,221.62 U/mL for the second one (Figure 2). For 14 (53.8%) of the first samples, the measured concentrations were higher compared to the second one; in 4 (15.4%), the titers were approximately equal in the second sample, and for 8 samples (30.8%), the values were higher in the second specimen. The average for the first sampling was 658.81 U/mL, and for the second one, the average was 540.58 U/mL.

For 23 mothers (88.5%), the anti-RBD IgA values were approximately 5-times higher than the IgG in the same sample, and 3 participants displayed higher values for IgG compared to IgA. While for two of these last women, no particular features could be recorded, it is worthwhile to note that the last case had the oldest baby within the study and generated extreme values. We have not considered this case as an outlier, since a reproducible pattern of extremely high concentrations could be observed for both IgA and IgG.

The variables’ distribution was verified using the Kolmogorov–Smirnov test. For the first IgA sample values, and for the babies’ and mothers’ ages, the distribution of the variable per group was normal (*p* > 0.05). For the normally distributed variable groups, we correlated the IgA value for the first sampling, the mother’s, and the baby’s age using the Pearson correlation. According to the Pearson test, the result of the IgA value for the first sampling and the baby’s age-correlation is statistically significant (*p* = 0.002), and there is a strong correlation between the anti-RBD IgA values and the babies’ age, the correlation coefficient result being r = 0.573. The anti-RBD IgA variables did not correlate to the mother’s age (*p* = 0.551) (Table 1).

**Figure 3:**
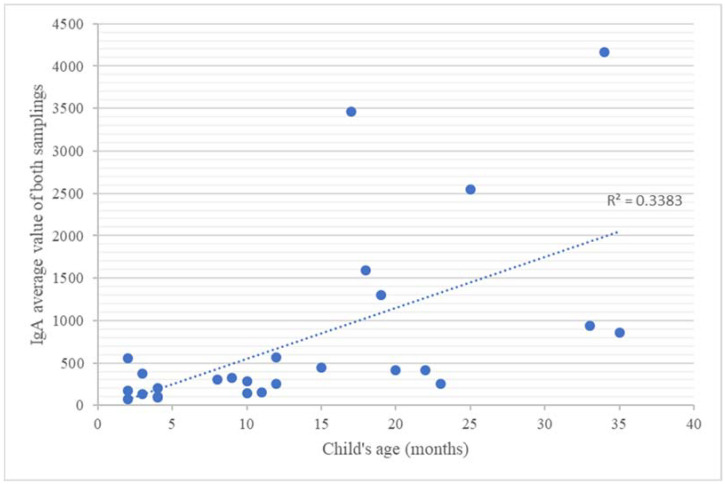
IgA average value of both samplings according to each infant’s age.

**Figure 4:**
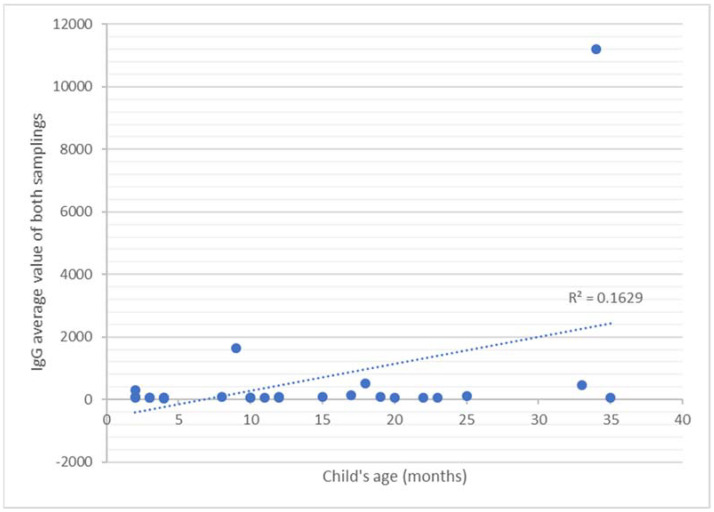
IgG average value of both samplings according to each infant’s age.

For the other variables, we used the Spearman test; therefore, we correlated the results of the second anti-RBD IgA sample and the results of anti-RBD IgG from both samples with the baby’s and mother’s ages, with the baby’s rank, and with the mother’s pathological history. The correlations of both IgA and IgG values with the child’s age were positive (*p* < 0.05) (Table 2): a strong relationship (r = 0.68) emerged between the anti-RBD IgA values and the child’s age (Figure 3), while a medium correlation between the anti-RBD IgG values and the child’s age could be noticed (r = 0.34 for the first collection and r = 0.32 for the second sampling) (Figure 4). The other parameters, such as the rank of the babies’ and the mothers’ ages, did not correlate with the amount of antibodies excreted in the breast milk. All results are presented in Table 2.

For group statistics, we compared the means of IgA and IgG values using the independent sample t-test, and we grouped the antibodies values, as dependent variables, with the type of the vaccine, the thyroiditis history, and the rank of the baby, as independent variables. We verified if there were any differences regarding the immunoglobulin excretion in breast milk in mothers vaccinated with the mRNA-1273 vaccine and the ones vaccinated with the BNT162b2 vaccine. The *p*-value of Levene’s test was higher than 0.05 for all the immunoglobulin variables. For all of the variables the *p*-value was higher than 0.05. The group statistics t-test for the immunoglobulins’ variables in mothers with autoimmune thyroiditis and without the disease showed a Levene’s *p*-value lower than 0.05, so we followed the conclusion of the *p* results. All of these *p* results were higher than 0.05, meaning that there is no significant difference between the mothers with or without thyroiditis regarding the excretion of milk antibodies. Another group t-test verified if there was any discrepancy in the antibody excretion depending on the child’s ranking. The Levene’s *p*-values were <0.05 and >0.05 for the anti-RBD IgA variables and for the anti-RBD IgG variables, respectively. We further noticed that the *p*-values for the anti-RBD IgA were smaller than 0.05, while for the anti-RBD IgG, they were higher than 0.05. Regarding the anti-RBD IgG values, no differences were observed among the children’s rank.

Anti-RBD IgA and IgG were detected in all the breast milk samples. We used the paired-samples t-test to compare the scores of two pair-variables, such as the results of the immunoglobulin testing from the first sampling and from the second one, or the results of anti-RBD IgA and IgG concentration measurements. The differences of the two samplings were not significant. The paired-samples t-test showed that there were no significant differences between the results of the anti-RBD IgA (*p* = 0.988) (Figure 1) or of the anti-RBD IgG (*p* = 0.284) of the two samplings (Figure 2).

In most of the cases, the anti-RBD IgA titer was much higher than the anti-RBD IgG value (*p* = 0.013). According to the paired-samples t-test, there are significant differences between the two groups, meaning that the anti-RBD IgA value is significantly higher compared to the anti-RBD IgG one. Overall, the specific IgA titer was only about five-times higher than IgG (mean anti-RBD IgA = 639.077 U/mL vs. mean anti-RBD IgG = 175.124 U/mL). 

## 3. Discussion

Although the anti-SARS-CoV-2 vaccinated adult population tends to increase steadily, the problem of unvaccinated children and, especially, of the unvaccinated babies is of major concern. As they tend to develop asymptomatic forms, they might represent an important reservoir of infection. Breastfed newborns might achieve some protection against SARS-CoV-2 infection if the ingested milk originates from vaccinated mothers. The human milk secretion is able to supply roughly 50-times more antibodies than those administered to a patient with hypogammaglobulinemia [37]. As the newborn is deficient in IgA, the particular class that protects mucosal membranes, it is not surprising that the overwhelmingly dominant isotype in milk is represented by the locally produced secretory IgA, which is meant to protect the infants from various pathogens [23,38,39,40]. Furthermore, breast feeding was shown to have an important impact upon the development and adjustment of the immune system of the neonate [38,40]. It has been long suggested that the vaccination of mothers might be an important modality to further boost the immunity of the breastfed infant via secretory IgA [23], but little attention was given to other immunoglobulin isotypes.

In order to see if some factors, such as the mothers’ and infants’ ages, parity, or vaccine type, may correlate with the amount of mother’s milk immunoglobulins delivered to infants during breastfeeding, we performed a detailed statistical analysis which included multiple correlation tests and group statistics.

The statistical analysis was performed with version 20 of the IBM SPSS platform. The Kolmogorov–Smirnov test revealed the distribution of the variables per group. We used the Spearman test for the variables which were not normally distributed according to the Kolmogorov–Smirnov test (*p* < 0.05), and the Pearson test for those which were normally distributed.

The results of all the immunoglobulin values were positively correlated with the child’s age (for the correlation with the first IgA sample, *p* = 0.002, for the second IgA sample, *p* = 0.000, for the first IgG sample, *p* = 0.046, and for the second IgG sample, *p* = 0.013), with a strong correlation between the anti-RBD IgA titers and the child’s age (r = 0.57 for the first sample and r = 0.68 for the second sample), while only a medium correlation (r = 0.34 for the first sampling and r = 0.32 for the second sampling) emerged between the child’s age and the anti-RBD IgG values. Taking into account the fact that the value of the correlation coefficient is positive, we can conclude that the values of anti-RBD IgA and IgG were directly proportional to the age of the child. This actually shows that the immune response expression in the breast milk directly depends on the period of breastfeeding: the longer the breastfeeding period, the higher the amount of milk immunoglobulins. These findings are supported by the results of Czosnykowska-Łukacka et al. [31], who showed that, during the first year of lactation, the concentrations of anti-RBD IgA and IgG were lower than in the second year, or the results of Ramirez et al. [15], showing as well that the antibody concentrations in the milk of mothers who were breastfeeding for 24 months were significantly higher than mothers who were breastfeeding for less than 24 months. Thus, in view of the high concentration of immunologically important compounds present in human milk, prolonged lactation should be strongly supported [31].

On the other hand, no correlation between the mothers’ age and the anti-RBD IgA titer (*p* > 0.05) could be found, suggesting that age does not predetermine or influence the anti-RBD IgA secretion in breast milk. This conclusion is in line with data published by Bachour et al. [41], showing that the human milk concentrations of proteins and secreted immunoglobulin A (sIgA) were not affected by the age of the mothers. Other parameters, such as the rank of the baby or the vaccine type, do not seem to influence the titer of the antibodies excreted in breast milk, leading to the conclusion that the breast milk antibody secretion is not influenced by these factors.

However, our results are in contradiction with the data published by Golan et al. [16], as they found a weak but significant negative correlation between infant age and milk anti-RBD IgA levels. Furthermore, twenty-five percent of the women in their cohort had no detectable levels of anti-RBD IgA in their milk 4–10 weeks after the second dose. This discrepancy might be explained by different characteristics of the study group, mostly in terms of infants’ age and collection intervals.

For group statistics, we compared the means of the variables using the independent sample t-test, and we grouped the antibodies values, as dependent variables, with the type of the vaccine or the rank of the baby, as independent variables. We verified if there were any differences regarding the immunoglobulin’s secretion in breast milk in mothers vaccinated with the mRNA-1273 vaccine and the ones vaccinated with the BNT162b2 vaccine. For all of the variables, the *p*-value was higher than 0.05. We could conclude that there was no significant difference between the mothers vaccinated with the mRNA-1273 vaccine vs. the BNT162b2 vaccine regarding the immunoglobulin’s level excreted in breast milk. Fox et al.’s [17] research led to the same conclusion: that no differences were detected in milk antibody titers between the groups of participants receiving each vaccine. 

Another group statistic t-test verified if there was any discordance in the antibody secretion depending on the child’s ranking. We observed that the *p*-values for the anti-RBD IgA were smaller than 0.05, and for the anti-RBD IgG, were higher than 0.05. That means that for the anti-RBD IgA variables there is a significant *p*, and they tend to be higher in breastfeeding of the first newborns. In conclusion, first-born babies receive a higher intake of anti-RBD IgA through breast milk comparing with further ranks. Weaver et al. and Prentice et al. described a negative correlation between parity and IgA and immunoproteins, respectively [42,43]. Bachour et al. [41] observed that milk protein concentration seemed to decrease with the increase in parity number, but the difference was not statistically significant in their study. They also underlined that the parity number was associated with irregular, statistically nonsignificant variations in sIgA concentration. Although the findings are not similar, our results support the need for further investigation of the aspect, on a larger cohort. The fact that the older siblings receive more sIgA might be the key for the differences that occur between the older and the younger siblings’ health and development [41,44,45,46]. However, as far as the anti-RBD IgG values are concerned, no differences could be noticed among the children’s rank.

We have shown the presence of anti-RBD IgA and IgG antibodies in all the breast milk samples of our cohort, thus demonstrating that these antibodies may be passed through human milk to infants, in accordance with the data published by other groups as well [14,17,22,38]. It is important to notice that the differences of the IgA and IgG titer between the first and the second sampling were not significant. The paired-samples t-test showed us that there were no significant differences between the results of anti-RBD IgA (*p* = 0.988) and of anti-RBD IgG (*p* = 0.284) from both samplings, suggesting that the amount of the antibodies is preserved for at least 60 days in breast milk. Similar results were found by Young et al. [18]. They also found that, in their cohort study of lactating parents, SARS-CoV-2 mRNA vaccination response in human milk began to decline 90 days after the second vaccine dose. The data of Collier et al. also support our findings, as they conclude that vaccination elicited binding and neutralizing antibodies in breast milk, suggesting the possibility that newborns may be protected by maternal vaccination [33]. 

In 88.5% of our cases, the titer of anti-RBD IgA was about five-times higher than the anti-RBD IgG one (*p* = 0.013). The difference was statistically significant according to the result of the paired-samples t-test. The fact that the excreted IgA amount is higher than the IgG one in breast milk is supported by many other authors in studies of general antibody detection in breast milk [23,33,47,48,49,50]. However, it is important to notice that while the normal milk has approximately nine-times more IgA than IgG [32], this ratio is decreased for the milk of vaccinated mothers, suggesting an increased transfer of the IgG isotype. This finding brings further evidence to support the role of the human FcRn present in the mammary gland epithelial cells to return the IgG to the mother’s circulation [36]. In fact, the presence of IgG in the human milk seems to reflect that there is a limited FcRn expression and, thus, recycling capacity within this tissue. Hence, the more IgG the organism will produce, the more it will have the possibility to surpass the FcRn barrier and enter the milk secretion. This is of particular importance, since the ingested IgG, unlike IgA, can be further transported across the intestine barrier into the newborn circulation by the FcRn expressed within the small intestine and colon [51].

## 4. Material and Methods

### 4.1. Study Design and Participants

We conducted a prospective study aimed at analyzing the IgA and IgG anti-SARS-CoV-2 titers in the breast milk of 28 vaccinated lactating mothers, from January through July 2021. The inclusion criteria were: two doses of Pfizer–BioNTech (BNT162b2) or Moderna (mRNA-1273) vaccines, no COVID-19 history until sampling, any type of breastfeeding (e.g., breastfeeding a single child or more, breastfeeding directly or by pumped milk, any time from the onset of breastfeeding), any age of the infants. Two participants were excluded from the final analysis due to occurrence of SARS-CoV-2 infection before the second sampling. All participants were volunteers and they were enrolled after signing an informed consent. 

An online questionnaire was filled out by every participant. It consisted of: demographic data, the date of the child’s birth, breastfeeding details, parity, vaccine type, vaccination schedule, post-vaccination side effects, and medical history.

The final group consisted of 26 lactating mothers aged between 29 and 37 years old. All of them were vaccinated anti-SARS-CoV-2 with 2 doses of mRNA vaccine. They lived in urban areas and worked in different fields. Data regarding the study group are included in Supplementary Materials Table S1.

### 4.2. Collection of Samples

Lactating mothers provided 2 breast milk samples at 30 and 60 days after the second vaccine dose. The samples were self-collected at home, in sterile containers, by manual expression or breast pumping. The harvesting was performed in a relaxing, cozy environment, in order not to influence the evacuation rate and composition of the milk. About 1 mL of milk was collected by the participants, from the medium flow in each one of 3 tubes. The specimens were frozen immediately at −20° C and stored until analyzed. The shipping was performed in refrigerated boxes.

### 4.3. Analysis of Samples 

Prior to processing, human milk samples were thawed and brought to room temperature. After spinning at 5000 g for 25 min at 4° C, the lipid ring that formed at the top of the tube was removed and the skimmed milk was transferred to a new tube for antibody assessment.

Reactive IgA and IgG antibodies anti-RBD, part of the spike S1 protein subunit of SARS-CoV-2 virus, were detected and quantified by a sandwich enzyme-linked immunosorbent assay (ELISA) (TestLine Clinical Diagnostics, Czech Republic, Catalog number: CoRA96-EIA COVID-19 RBD IgA, CoRG96 - EIA COVID-19 RBD IgG), with a test specificity of 98.86% and 99.15% for the IgA and IgG, respectively, according to the manufacturer. The sensitivity of the test is of 96.6% and 99.9% for IgA and IgG, respectively.

The method was, at first, validated for human milk by testing various concentrations and dilutions of the sampled breast milk. We were, thus, able to conclude that a 1:10 dilution is optimal. All specimens were diluted 1:10 in the sample diluent provided by the kit (25 µL of human milk in 225 µL diluent buffer) and mixed by vortexing. The samples were processed in accordance with the manufacturer’s indications. The optical densities were measured with a TECAN Infinite 200 photometer at a 450-nm wavelength and the results were processed with the Magellan software. The cutoff value provided by the manufacturer was 5 U/mL.

### 4.4. Ethical Principles

The study complied with the ethical principles stated by the World Medical Association Declaration of Helsinki, regarding medical research involving human subjects. The study was approved by the Commission of Ethics of Research from the University of Medicine and Pharmacy “Grigore T. Popa” Iași, Romania (IRB number: 99/2021).

### 4.5. Statistical Analysis

All data were analyzed using the IBM SPSS statistical software version 20. The Kolmogorov–Smirnov test was used to assess the distribution of the variables. A *p* > 0.05 underlined a normal distribution and a *p* < 0.05 underlined that the variables are not normally distributed. Following this step, the Pearson correlation test was used for the normally distributed variables and the Spearman correlation test for the others. *p* < 0.05 was considered as significant; the ranking of the correlation coefficient between variables was detected by the r result as following: if the result is between 0–0.29,it suggests a poor connection; if it is between 0.3–0.49, it underlines a medium connection, and if it is in the 0.5–1 interval, it shows a strong connection. For group statistics, we compared the groups using the independent sample t-test and paired-samples t-test. *p* < 0 for the Levene’s test and *p* < 0.05 were considered as significant. The conclusions of the studies were supported by the results of the statistics tests.

## 5. Conclusions

In conclusion, the mRNA vaccine’s protection can be delivered to infants via passive immunity through human breast milk of vaccinated mothers. This anti-SARS-CoV-2 antibody protection is supplied by anti-RBD IgA mucosal immunity of the gastrointestinal tract, the main portal of entry for microorganisms in babies, and by anti-RBD IgG. The anti-RBD IgA titer was five-times higher than the anti-RBD IgG amount. The anti-RBD IgA and IgG titers did not decrease after 60 days. The antibody response is directly proportional with the breastfed child’s age. The amount of anti-RBD IgA decreases with the baby’s rank. The antibody response does not depend on the vaccine type, nor on the mother’s age.

## 6. Limitations

Our study has several shortcomings. We could only consider 26 lactating mothers; hence, the statistical conclusions might be somehow biased, the size of the study group being somehow similar to that reported by others [31]. However, we are confident that our conclusions are reasonably sound, as many of them are supported by other authors as well. We were not able to assess the serum levels of the anti-RBD antibodies. This would have been extremely useful, especially for understanding the behavior of the IgG antibodies, the major immunoglobulin isotype in the serum. The data characterizing the IgG antibodies levels of the women with autoimmune thyroiditis were not available to us, as it would have been useful to evaluate if a high level of IgG autoantibodies might hinder, in any way, the transfer of the IgG antibodies generated by the vaccine.

Subsequent studies may examine the period from the onset of breastfeeding when the most effective antibody transfer happened, to try to synchronize the mother’s vaccination with the transfer of passive immunity.

## Figures and Tables

**Table 1:** *p*-values according to the Pearson correlation test, calculated for the correlations of the first IgA sample, the child’s age, and the mother’s age.

*p*-Value	First Sample IgA	Child’s Age	Mother’s Age
First sample IgA		0.002	0.551
Child’s age	0.002		0.035
Mother’s age	0.551	0.035	

**Table 2:** *p*-values according to the Spearman correlation test, calculated for the correlation of the IgA and IgG values and the child’s age and rank and the mother’s age.

*p*-Value	First Sample IgA	Second Sample IgA	Mean IgA Value	First Sample IgG	Second Sample IgG	Mean IgG Value
Child’s Age		0.000	0.000	0.046	0.013	0.041
Child’s Rank	0.599	0.282	0.456	0.999	0.476	0.393
Mother’s Age		0.215	0.639	0.334	0.393	0.348

## Data Availability

The data presented in this study are available in Table S1.

## Supplementary Materials

The following supporting information can be downloaded at https://www.mdpi.com/article/10.3390/pathogens11030286/s1, Table S1. Data regarding the study group.
