# Supplementary material for: Anti-RBD IgA and IgG Response and Transmission in Breast Milk of Anti-SARS-CoV-2 Vaccinated Mothers
Source: Pathogens. 2022 Feb 24;11(3):286. doi: 10.3390/pathogens11030286 (PMC8952534; doi:10.3390/pathogens11030286)
Supplement: Supplementary file 1 [file pathogens-11-00286-s001.zip › pathogens-1561012-supplementary.pdf]

Table S1. Data regarding the study group

No	IgA 1st sample	IgA 2nd sample	IgG 1st sample	IgG 2nd sample	Vaccine	Date of vaccination	Date of child birth	Child's age at the time of vaccination	Child's age at the time of 1st sampling	Child's age at the time of 2nd sampling	Parity	Mother's age
1	183.12	471.76	2953.6	328.51	Pfizer	24.04.2021	5.01.2021	9l	10	11	1	30
2	271.75	235.31	45.313	46.656	Pfizer	17.01.2021	12.02.2019	23l	24	25	1	31
3	109.28	94.605	22.962	39.435	Moderna	16.03.2021	27.10.2020	4l	5	6	1	38
4	84.872	64.435	523.59	72.057	Pfizer	10.02.2021	1.12.2020	2l	3	4	2	33
5	2621	2465	156.09	68.026	Pfizer	10.02.2021	10.01.2019	25l	26	27	1	31
6	277.69	467.08	58.678	52.169	Pfizer	1.02.2021	20.10.2020	3l	4	5	2	36
7	181.11	158.4	49.191	51.241	Pfizer	18.02.2021	1.12.2020	2l	3	4	2	33
8	946.33	931.49	783.22	146.6	Pfizer	5.02.2021	31.03.2018	33l	34	35	1	31
9	510.55	624.02	56.499	67.349	Pfizer	2.02.2021	3.02.2020	12l	13	14	2	34
10	992.06	720	71.921	56.506	Pfizer	7.02.2021	18.02.2018	35l	36	37	1	31
11	1080	1511.3	59.776	102.28	Pfizer	25.01.2021	3.06.2019	19l	20	21	1	35
12	171.4	243.45	59.776	55.635	Pfizer	21.02.2021	13.10.2020	4l	5	6	2	34
13	147.35	137.8	55.311	54.391	Pfizer	11.02.2021	10.04.2020	10l	11	12	1	32
14	151.37	412.04	49.939	77.774	Pfizer	7.04.2021	4.06.2020	10l	11	12	1	32
15	4161.707	4159.966	11206.34768	11221.61956	Pfizer	3.04.2021	28.06.2021	34l	35	36	1	32
16	1271.5	1916.6	172.73	872.17	Pfizer	25.04.2021	04.10.2019	18l	19	20	1	29
17	467.31	371.09	74.268	59.873	Pfizer	28.01.2021	14.05.2019	20l	21	22	2	35
18	185.95	331.97	70.166	97.388	Pfizer	18.05.2021	16.05.2020	12l	13	14	2	35
19	701.26	407.89	90.618	74.101	Moderna	12.03.2021	16.01.2021	2l	3	4	3	37
20	139.92	166.01	59.484	51.17	Pfizer	29.02.2021	20.03.2020	11l	12	13	1	34
21	98.788	97.168	54.219	69.018	Pfizer	31.03.2021	21.11.2020	4l	5	6	1	32
22	134.28	135.19	52.308	53.624	Pfizer	14.02.2021	21.10.2020	3l	4	5	1	30
23	350.62	485.84	82.721	57.669	Pfizer	9.02.2021	13.04.2019	22l	23	24	1	30
24	699.82	197.47	81.983	85.832	Pfizer	9.02.2021	25.10.2019	15l	16	17	1	35
25	404.82	197.24	86.606	63.9	Moderna	14.03.2021	13.07.2020	8l	9	10	2	36
26	3805.148	3123.4	151.75	130.14	Pfizer	30.01.2021	12.08.2019	17l	18	19	1	34
